# Attraction Propagation: A User-Friendly Interactive Approach for Polyp Segmentation in Colonoscopy Images

**DOI:** 10.1371/journal.pone.0155371

**Published:** 2016-05-18

**Authors:** Ning Du, Xiaofei Wang, Jianhua Guo, Meidong Xu

**Affiliations:** 1 School of Mathematics and Statistics, Northeast Normal University, 130024 Changchun, China; 2 Zhongshan Hospital Fudan University, Fudan University, 200032 Shanghai, China; 3 Key Laboratory of Applied Statistics of MOE, Northeast Normal University, 130024 Changchun, China; University Hospital Llandough, UNITED KINGDOM

## Abstract

The article raised a user-friendly interactive approach-Attraction Propagation (AP) in segmentation of colorectal polyps. Compared with other interactive approaches, the AP relied on only one foreground seed to get different shapes of polyps, and it can be compatible with pre-processing stage of Computer-Aided Diagnosis (CAD) under the systematically procedure of Optical Colonoscopy (OC). The experimental design was based on challenging distinct datasets that totally includes 1691 OC images, and the results demonstrated that no matter in accuracy or calculating speed, the AP performed better than the state-of-the-art.

## Introduction

The colorectal cancer now has been certified as the third most common cancer throughout the world. There will be close to 2 million new cases annually, and as the Top 4 disease mortality, in each year, around 600 thousands of people died from it [1, 2]. According to statistics, most cases of colorectal cancer are pathological changed from colorectal polyp, therefore, to identify the types of colorectal polyp at early stage and to target it to solve the problem may reduce the death rate, and even cure patients [3, 4]. Colonoscopy as the golden standard method has been widely used in screening and colorectal polyp diagnosis [5]. However, even a well-trained experienced medical staff still needs to spend tremendous time on suffering the optical colonoscopy (OC) images and colorectal polyp analysis. So to combine the Computer-Aided Diagnosis (CAD) system into the screening and colorectal polyp diagnosis could alleviate the burden of medical staffs’ routing works.

**Fig 1:**
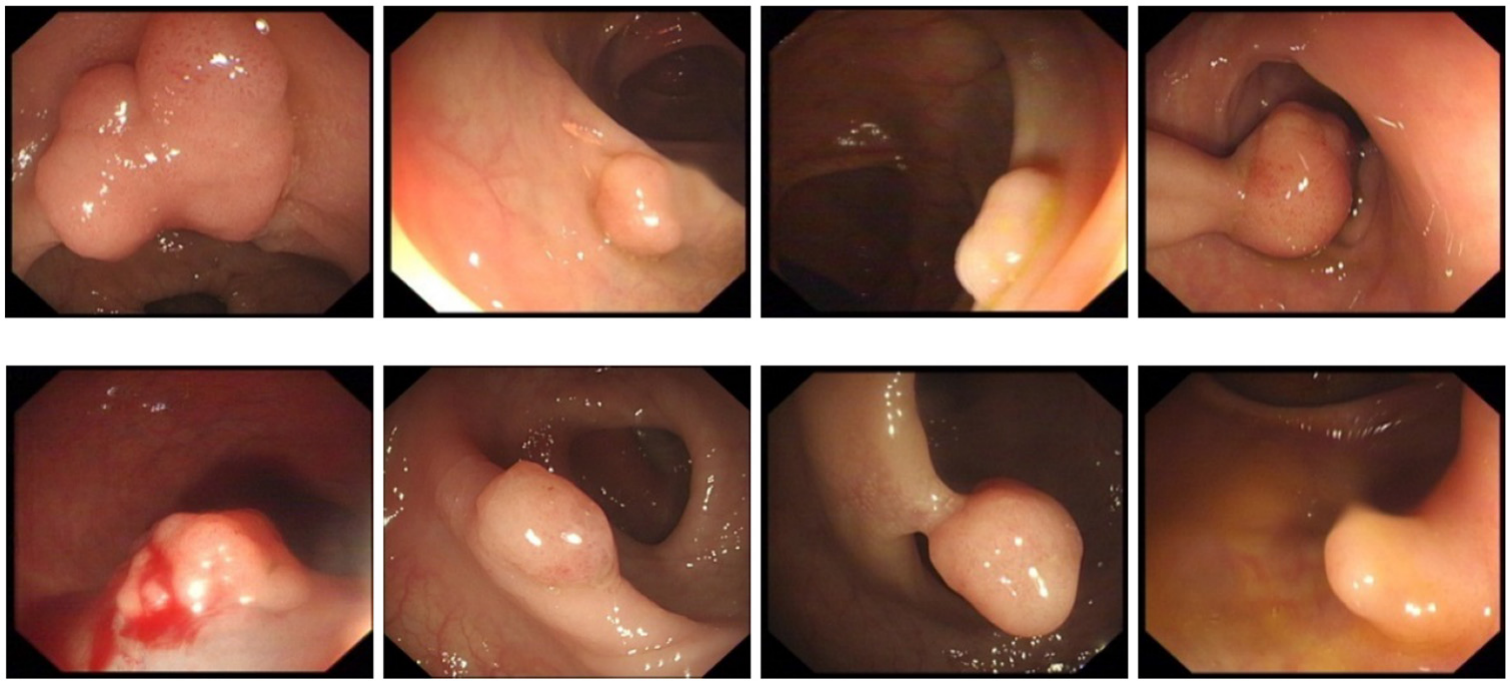
Optical colonoscopy (OC) images with polyp.

Generally, a CAD system based on OC image consists of several procedures: image preprocessing, segmentation, feature selection, and classification. Among those procedures, image segmentation is a key procedure in the CAD system. Accurate segmentation of abnormal regions can improve the performance of the CAD system significantly [6]. Because current diagnoses based on endoscope have no calibration and are more operator-dependent than other diagnoses(such as X-ray or MRI), we may encounter a high inter-observer variation among different clinicians as shown in Fig 1. The variation and uncertainty makes the segmentation of OC images become a challenging task.

Numerous methods have been proposed for automatic segmentation, such as Fuzzy C-means [7], Mean Shift [8], Spectral Clustering [9], Quick Shift [10], and Entropy-rate clustering (ERC) [11]. These methods do not require the guidance from users to specify the segmented regions and they select an appropriate set of features according to different criteria. These features are helpful for merging regions with the same property and for identifying regions with different properties. Although these automatic methods produce some satisfactory segmentation results for OC images (as shown in Section 3), they are still impacted on by some factors producing an inaccurate segmentation, such as blood vessels, bubbles, and excreta, and the various categories of polyps, which can be flat, protruded, or depressed in shape [12–16]. To solve the problems above, Gross et al. [17] use a template matching approach which exploits the ellipse boundary shape to segment polyps in OC images. But the restriction to the type of the template produces some unsatisfactory segmentations. Breieret al. [18] apply the Chan-Vese-Segmentation for extracting the boundary of polyps in OC images. Although their method can offer the flexibility to adapt to shape variation well without using edge information, the time cost is huge because of the involvement of a large amount of iterations. Bernal et al. [12] propose a method based on the depth of valleys image to realize the OC image segmentation effectively. However, in some cases, a fixed parameter is not available that works well for all OC images. Cong et al. [19] gives a super-pixel segmentation method for endoscopy images, while it suffers from over-segmentation with less robustness.

In recent years, interactive/semi-automatic methods for image segmentation have attracted much attention and they have become quite mature. In contrast to automatic segmentation methods, interactive methods require that some foreground and background pixels are marked manually (these marked pixels are known as seed points), before completing the segmentation of the remaining regions according to the seed points. Several popular interactive segmentation methods are available, including Active Contours (AC) [20–24], Lazy Snapping [25], Level Set (LS) [26–29], Graph Cuts (GC) [30, 31], GrabCut [32], Random Walks (RW) [33–35], and Shortest Paths (SP) [36]. Among these methods, the weighted graph method (such as GC, RW, SP) is a powerful tool for the interactive image segmentation. Previous studies [35, 37, 38] have demonstrated that this method can identify multiple objects simultaneously and performs well with different seeding strategies (such as equidistant seeds or strongly asymmetric seeds). Moreover, it is robust with high computational speed.

Although weighted graph method is valid for various types of image segmentation, it encounters small cut problems during image segmentation, especially when low numbers of foreground and background seeds are marked. The underlying cause of these problems lies in that the weighted graph or diffusion distance [37] is calculated only from adjacent information (such as Laplacian matrix) as solving the optimal problem of graph cuts, and the transition probabilities are difficult to propagate to the region far from the seeds. These existing problems greatly reduce the accuracy and quality of image segmentation. In recent years, the traditional weighted graph method has been extended to some new versions [37, 38]. In particular, Couprie et al. [37] suggest a unified framework for GC, RW, SP and Watersheds. By combining the RW with the Maximum Spanning Forest, their algorithm produces an improved segmentation of a higher speed. However, in some cases, this algorithm is also affected by the small cut problem in the same manner as the RW.

In this study, we propose an interactive segmentation algorithm called attraction propagation (AP) for OC image segmentation, which compensates for some of the shortcomings of interactive methods such as AC, LS, GC, and RW. We introduce an attraction scheme based on a shape probability region and reconstruct an image graph for segmentation. In contrast to existing interactive methods, this new implementation scheme AP can solve the small cut problem in an effective manner by boosting the transition probability of pixels in attraction region. When encountering weak boundary problems, AP also produces more accurate segmentation results than many other methods such as AC, LS, RW and PW.

Now we summarize the main contributions of this proposed method:

- We provide a feasible strategy for the initialization of only one foreground seed. This strategy can be very easily integrated into the current clinical diagnosis and OC image acquisition.
- We introduce a shape probability region to offer an attraction region effectively, and propose a flexible framework for polyp segmentation in OC images.
- We give a fast minimization algorithm for the OC image processing.
- We build an open database of 800 OC images for assessing the performance of segmentation algorithms.

The remainder of this paper is organized as follows. Section 2 describes the proposed AP algorithm. Section 3 presents the experimental results obtained after the OC image segmentation. Section 4 discusses the sensitivity of parameters about our algorithm. In Section 5, we summarize the proposed algorithm.

## Methods

In this section, we firstly give a user-friendly initialization of seed placement, and then introduce related works of weighted graph method. Secondly, we propose the attraction propagation algorithm with three forms. Finally, we provide some experimental examples to illustrate the effectiveness of our algorithms.

### The initialization of seed placement

**Fig 2:**
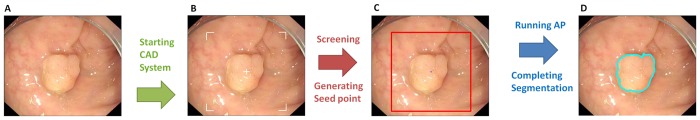
Overview of AP algorithm designed for CAD system. (A) Display screen of endoscopic diagnosis. (B) Display screen after starting the CAD system. (C) The seed placement automatic initialization base on white viewfinder in (B). (D) The segmentation result of AP.

To segment OC images through interactive approaches, the primary problem lies in how to handle the initialization of seed placement. For the conventional initialization of seeds, users need to provide a large amount of interactive operations, which are laborious, exhausting and hard to be integrated into the current clinical diagnosis. In this paper, we provide a feasible and convenient strategy for initializing seeds. A schematic view of this strategy is given in Fig 2. Fig 2B shows a picture with a viewfinder. The polyp can be captured in the frame formed by the viewfinder as shown in Fig 2C. AP will detect the contours of polyps according to the seeds in Fig 2C. Specially, when the polyp is in a fixed position of the image, AP can realize automatic segmentation.

### Related work

**Fig 3:**
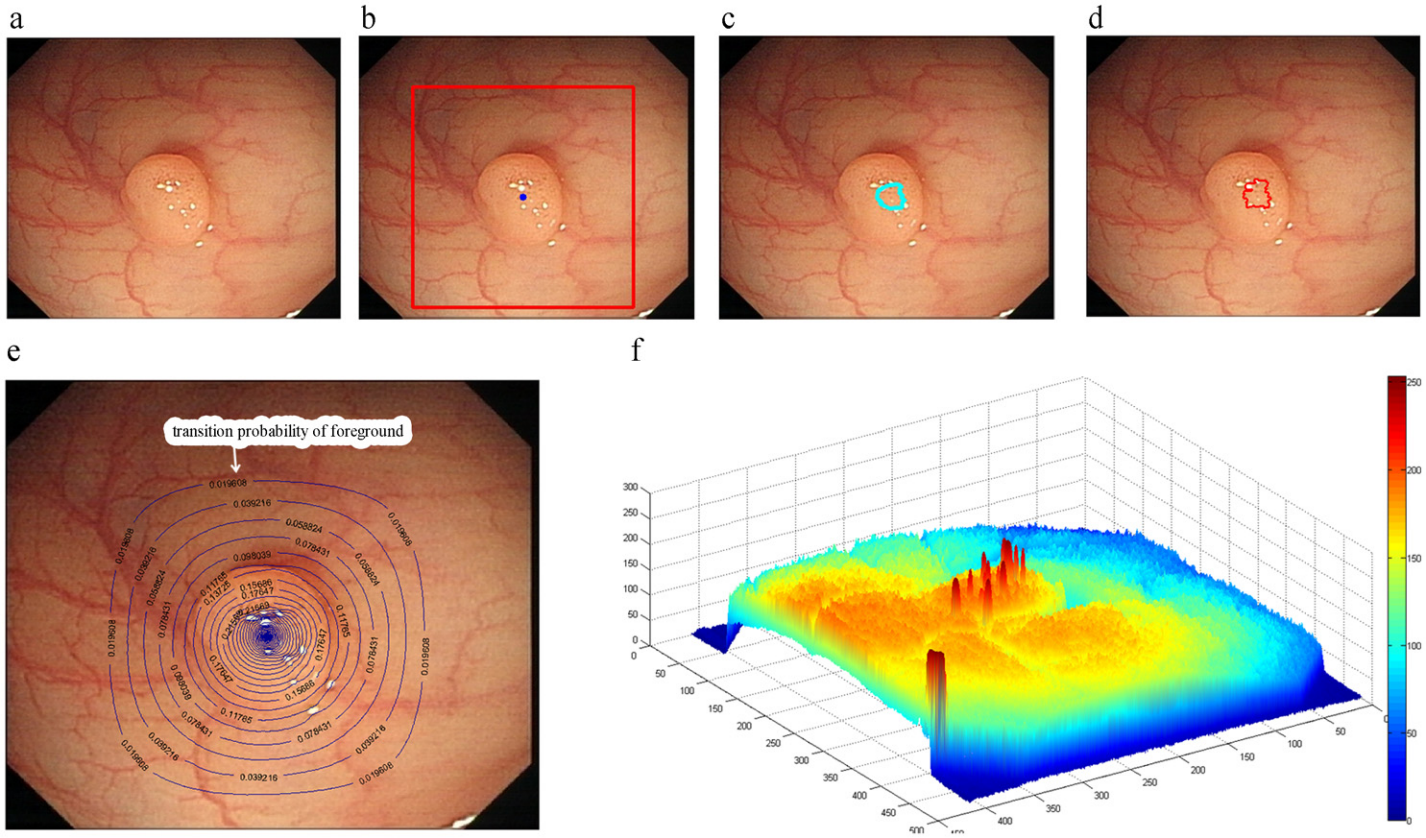
(a) original images; (b) seeds of foreground and background; (c) random walks’output; (d) power watershed’s output; (e) contour plot of transition probability of foreground obtained by random walks; (f) 3d representation of the greyscale OC image.

Given an image *I*, the segmentation problem can be formulated on an undirected and connected graph *G* = (*V*, *E*) [39], where vertices (nodes) *v* ∈ *V* and edges *e* ∈ *E* ⊆ *V* × *V*, and the cardinalities of *V* and *E* are |*V*| and |*E*|, respectively. An edge that spans two vertices *v*_*i*_ and *v*_*j*_ is denoted by *e*_*ij*_. A weighted graph assigns a weight to each edge. The weight of an edge, *e*_*ij*_, is denoted by *w*(*e*_*ij*_) or *w*_*ij*_, which is nonnegative (i.e., *w*_*ij*_ ≥ 0). The edge set *E* comprises pairs of pixels, which are neighbors in the image. A Gaussian function of the L1 distance is defined between pixels with different image intensities, for which the weight *w*_*ij*_ can be defined as

$$w_{ij}=exp\left(-\beta \left|g_{i}-g_{j}\right|\right),$$ (1)

where *g*_*i*_ indicates the image intensity at pixel *i* and *β* is a free parameter. In weighted graph scenario, image segmentation is considered on an image domain (for the case with two labels). Every unlabeled node is given a label according to different energy function. The labels of nodes decide a segmentation of graph. Among these methods, the RW and PW are two powerful and robust tools [34, 37], so we choose them in experiments for discussing and illustrating the effects of graph methods. Fig 3c and 3d show the segmentation results of RW and PW by choosing the seeds shown in Fig 3b. Under new strategy for initialization seed (see the previous subsection), these method may encounter the small cut problem.

**Fig 4:**
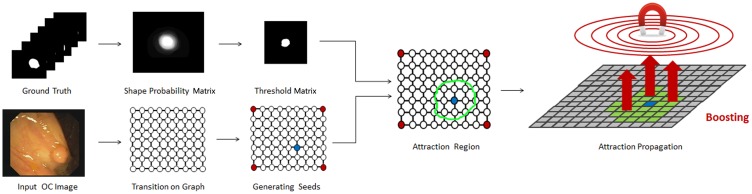
A schematic plot of the attraction propagation (AP).

To solve the small cut problem, we try to use the shape prior knowledge for enhancing the segmentation results. Actually, there are already several works discussing how to utilize the shape prior knowledge to increase the robustness and accuracy of medical image segmentation. For instance, Aslan et al. [40] use a 3D shape model obtained from a training data set to overcome any in homogeneity in CT images of bones. Chowdhury et al. [41] apply a probabilistic variation of the traditional graph cut algorithm and address the problem of segmentation of cerebral white matter from T1-weighted MRI data. To process cardiac MRI, Grosgeorge et al. [42] propose a segmentation method based on a statistical shape model obtained with a principal component analysis. Inspired by those works, we propose a new segmentation scheme called as AP which uses the linear system to give an optimization solution. By learning the shape priori-knowledge in OC databases, AP offers to determine attraction region and boost transition probabilities for nodes in the region. Fig 4 shows a schematic plot of our strategy, and the concrete attraction mechanics are introduced in the following subsections.

### The attraction propagation (AP)

In this subsection, we propose the attraction propagation (AP) to segment the input images. The attraction propagation term $\underset{}{\sum_{v_{i}\in A}D\left(a_{i},x_{i}^{c},\tau_{i}^{c}\right)}$ is introduced in the weighted graph method to control the transition probability. The new segmentation model is as

$${\text{argmin}}_{x}\left\{\sum_{v_{i}\in A}D\left(a_{i},x_{i}^{c},\tau_{i}^{c}\right)+\sum_{e_{ij}\in E}w_{ij}{\left(x_{i}^{c}-x_{j}^{c}\right)}^{2}\right\},$$ (2)

where the $\;x_{i}^{c}$ is the transition probability of category *c* and *c* represents the category. In our problem *c* = *f* or *c* = *b*, where *f* and *b* represent the foreground and the background respectively. A is a set of vertices in the attraction region (AR). We will give a detailed description on how to obtain the attraction region in the next subsection. In our model, $a_{i},\tau_{i}^{c}$ and *w*_*ij*_ are three adaptive parameters. The summand in the attraction propagation term can choose several forms according to various problem-specific domains. In this paper, we give three basic forms

Pearson distance [43]:

$$D_{P}\left(a_{i},\;x_{i}^{c},\tau_{i}^{c}\right)=a_{i}{\left(x_{i}^{c}-\tau_{i}^{c}\right)}^{2}/2\tau_{i}^{c},$$ (3)

Inner product:

$$D_{In}\left(a_{i},\;x_{i}^{c},\tau_{i}^{c}\right)=a_{i}x_{i}^{c}\tau_{i}^{c},$$ (4)

L2 norm square:

$$D_{L2}\left(a_{i},\;x_{i}^{c},\tau_{i}^{c}\right)=a_{i}{\left(x_{i}^{c}-\tau_{i}^{c}\right)}^{2}.$$ (5)

Basing on the AP model (2), we can compute the transition probability $\;x_{i}^{c}$, and assign a label to every unlabeled node for getting the final segmentation. By expanding the attraction propagation and the weighted graph terms, we rewrite them in the matrix forms

$$D_{P}\left(A,x,\tau \right)=\underset{}{\sum_{v_{i}\in A}D_{P}\left(a_{i},x_{i}^{c},\tau_{i}^{c}\right)=\frac{1}{2}x^{T}\left(A\tau^{-1}I^{T}\cdot I\right)x-x^{T}\left(A\tau^{-1}I^{T}\cdot I\right)\tau +\frac{1}{2}\tau^{T}\left(A\tau^{-1}I^{T}\cdot I\right)\tau ,}$$ (6)

$$D_{In}\left(A,x,\tau \right)=\underset{}{\sum_{v_{i}\in A}D_{In}\left(a_{i},x_{i}^{c},\tau_{i}^{c}\right)=x^{T}A\tau ,}$$ (7)

$$D_{L2}\left(A,x,\tau \right)=\underset{}{\sum_{v_{i}\in A}D_{L2}\left(a_{i},x_{i}^{c},\tau_{i}^{c}\right)=x^{T}Ax-2x^{T}A\tau +\tau^{T}A\tau ,}$$ (8)

$$G\left(W,x\right)=\underset{}{\sum_{e_{ij}\in E}w_{ij}{\left(x_{i}^{c}-x_{j}^{c}\right)}^{2}={\left(Kx\right)}^{T}W\left(Kx\right)=x^{T}Lx,}$$ (9)

where ⋅ denotes an element-by-element multiplication and *τ*^−1^ is an element-by-element reciprocal of a column vector *τ*. I is a column vector where each element is one.

### The attraction region and some details of AP

**Fig 5:**
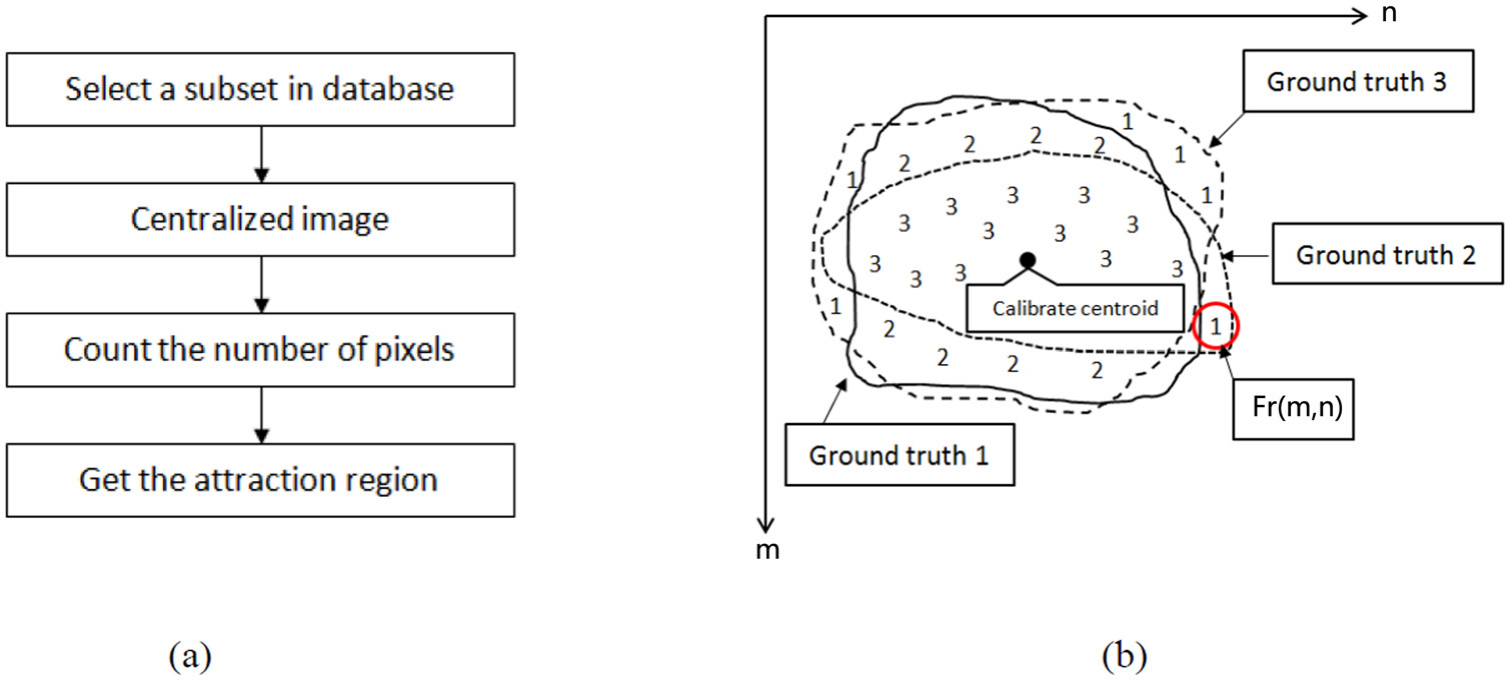
(a) A flow chart of the shape prior region; (b) Illustration of generating the shape probability matrix.

In this subsection, we first illustrate how to obtain the attraction region A from the shape prior. And then we give the detailed description of formulas (6)–(9). A flow chart of the attraction region is shown in Fig 5a and a detailed procedure is given as follows:

Step 1. Select a subset in the OC ground truth image database for generating the attraction region.
Step 2. Calculate the centroids of ground truth images and calibrate these centroids to the centre position.
Step 3. Count the number *Fr*(*m*, *n*) of pixels in the overlapping regions of calibrated images. Normalize the number *Fr*(*m*, *n*) by
  $$SP\left(m,n\right)=\frac{Fr\left(m,n\right)}{Ns},$$ (10)
  Where *m* and *n* represent the coordinates, *Ns* is the number of ground truth images. We call *SP*(*m*, *n*) as the shape probability matrix (see Fig 4).
Step 4. Choose a threshold matrix by
  $$T\left(m,n\right)=\{\begin{array}{cc} SP\left(m,n\right) & if\;SP\left(m,n\right)\geq \Gamma ,\\ 0 & otherwise, \end{array}$$ (11)
  where *Γ* is a threshold and 0 ≤ *Γ* ≤ 1. We use the non-zero region in the threshold matrix as the shape prior region (see Fig 4). An illustrative example of generating the shape probability matrix is shown in Fig 5b for *Ns = 3*.

Now we illustrate concrete definitions of symbols in formulas (6)–(8). Given one foreground seed, we introduce an attraction region  A generated by shifting the shape prior region onto the seed such that the position of seed is same as the centroid of the region (see Fig 4).

The matrix *A* is a diagonal |*V*| × |*V*| attraction propagation incidence matrix and defined as follows

$$A\left(i,j\right)=\{\begin{array}{cc} a_{i} & if\;i=j\;and\;v_{i}\in A\;,\\ 0 & otherwise. \end{array}$$ (12)

**Fig 6:**
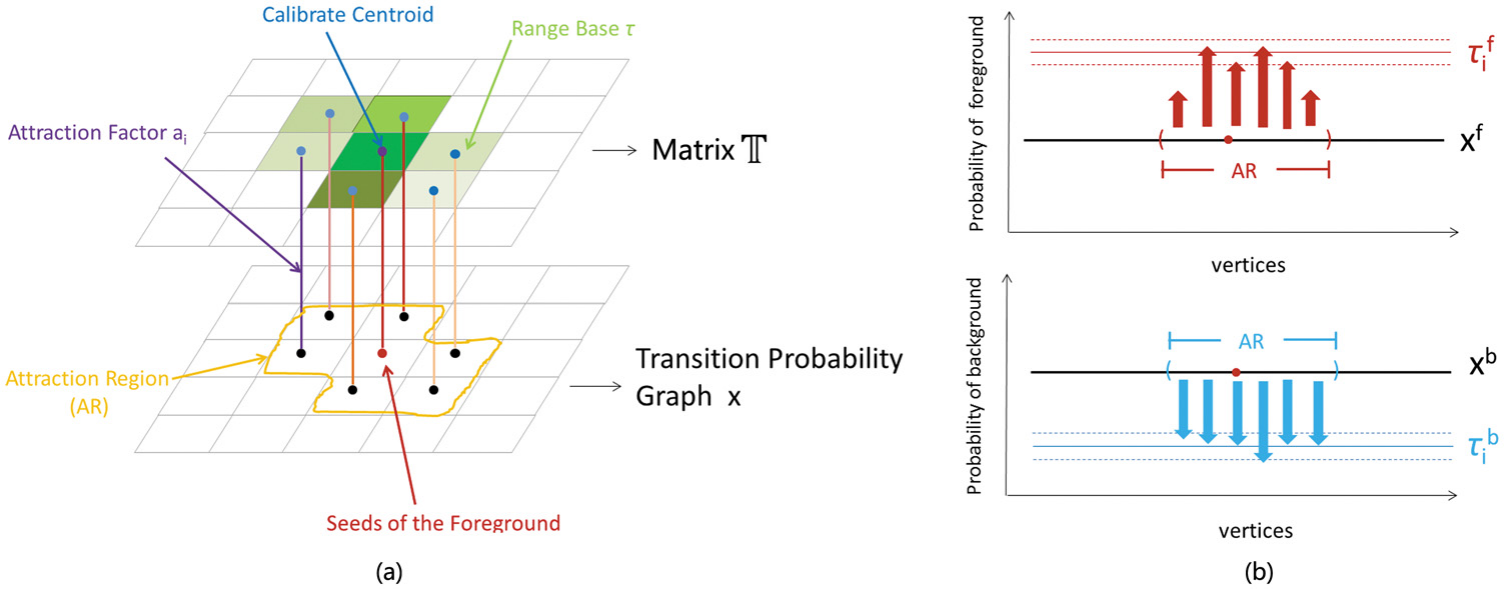
(a) An intuitive example to demonstrate matrix  T. (b) the schematic plot of computing the probability of foreground and background. Red represents seeds of the foreground and the centroid of the attraction region.

The attraction factor *a*_*i*_ is used to adjust the attraction intensity of pixels in the  A. In this work, the attraction factor *a*_*i*_ = *exp*(−|*g*_*i*_ − *g*_*s*_|), where *g*_*s*_ is the image intensity of seed and *s* is a foreground seed. In formulas (6)–(8), the range base *τ* in *R*^|*V*|^ for controlling the scope of probabilitiesis defined by

$$\tau \left(i\right)=\{\begin{array}{c} \tau_{i}^{c}=\;Vec\left(T\right)\left(i\right)\;if\;v_{i}\in A,\;c=f,\\ \tau_{i}^{c}=\;1-Vec\left(T\right)\left(i\right)\;if\;v_{i}\in A,\;c=b,\\ \;0\;otherwise, \end{array}$$ (13)

where T is a matrix with the same size of input image. Fig 6a gives an intuitive example to demonstrate matrix  T. It is constructed by shifting the threshold matrix *T* in formula (11) onto the foreground seed such that the center coordinate of *T* is same as the position of seed. *Vec*(·) operator stacks the entries of a 2-dimensional matrix into a column vector. We hope to boost the probabilities of pixels in the attraction region when computing the probabilities in the foreground, and depress those probabilities in the attraction region when considering the transition probabilities in the background as shown in Fig 6b.

In formula (9), the |*E*| × |*V*| edge-node incidence matrix *K* is defined as

$$K_{e_{ij},v_{k}}=\{\begin{array}{l} +1\\ -1\\ 0 \end{array}\begin{array}{r} if\;i=k,\\ if\;j=k,\\ otherwise, \end{array}$$ (14)

and the |*E*| × |*E*| matrix *W* is a constitutive diagonal matrix with the square weights of each edge along the diagonal. Furthermore, the Laplacian matrix *L* of the graph is defined as follows

$$L\left(i,j\right)=\{\begin{array}{c} \sum_{k}w_{ik}\;if\;i=j,\\ -w_{ij}\;if\;v_{i}\;and\;v_{j}\;are\;adjacent\;nodes,\\ 0\;otherwise. \end{array}$$ (15)

Now we give the detailed description of formulas (6)–(9).

Pearson distance:

$$\begin{array}{lll} D_{P}\left(A,x,\tau \right)+G\left(W,x\right) & = & x^{T}\left(\frac{1}{2}A\tau^{-1}I^{T}\cdot I\right)x-x^{T}\left(A\tau^{-1}I^{T}\cdot I\right)\tau +\tau^{T}\left(\frac{1}{2}A\tau^{-1}I^{T}\cdot I\right)\tau +\;x^{T}Lx\\ & = & \left[x_{M}^{T}\;x_{U}^{T}\right]\left[\begin{array}{cc} {\mathbb{P}}_{M} & B\\ B^{T} & {\mathbb{P}}_{U} \end{array}\right]\left[\begin{array}{c} x_{M}\\ x_{U} \end{array}\right]-\left[x_{M}^{T}\;x_{U}^{T}\right]\left[\begin{array}{cc} 2A_{M} & 0\\ 0 & 2A_{U} \end{array}\right]\left[\begin{array}{c} \tau_{M}\\ \tau_{U} \end{array}\right]+\left[\tau_{M}^{T}\tau_{U}^{T}\right]\left[\begin{array}{cc} A_{M} & 0\\ 0 & A_{U} \end{array}\right]\left[\begin{array}{c} \tau_{M}\\ \tau_{U} \end{array}\right]\\ & = & x_{M}^{T}{\mathbb{P}}_{M}x_{M}+2x_{U}^{T}B^{T}x_{M}+x_{U}^{T}{\mathbb{P}}_{U}x_{U}-2x_{M}^{T}A_{M}\tau_{M}-2x_{U}^{T}A_{U}\tau_{U}+\tau_{M}^{T}A_{M}\tau_{M}+\tau_{U}^{T}A_{U}\tau_{U}. \end{array}$$ (16)

We denote $\;A=\frac{1}{2}A\tau^{-1}I^{T}\cdot I$ and L+A as ℙ, which is partitioned into marked (seed nodes) and unmarked (unseeded nodes) blocks.

$$\mathbb{P}=\left[\begin{array}{cc} {\mathbb{P}}_{M} & B\\ B^{T} & {\mathbb{P}}_{U} \end{array}\right].$$ (17)

Inner product:

$$\begin{array}{lll} D_{In}\left(A,x,\tau \right)+G\left(W,x\right) & = & x^{T}A\tau +\;x^{T}Lx\;=\left[x_{M}^{T}\;x_{U}^{T}\right]\left[\begin{array}{cc} A_{M} & 0\\ 0 & A_{U} \end{array}\right]\left[\begin{array}{c} \tau_{M}\\ \tau_{U} \end{array}\right]+\left[x_{M}^{T}\;x_{U}^{T}\right]\left[\begin{array}{cc} L_{M} & B\\ B^{T} & L_{U} \end{array}\right]\left[\begin{array}{c} x_{M}\\ x_{U} \end{array}\right]\\ & = & x_{M}^{T}L_{M}x_{M}+2x_{U}^{T}B^{T}x_{M}+x_{U}^{T}L_{U}x_{U}+x_{M}^{T}A_{M}\tau_{M}+x_{U}^{T}A_{U}\tau_{U}. \end{array}$$ (18)

L2 norm square:

$$\begin{array}{lll} D_{L2}\left(A,x,\tau \right)+G\left(W,x\right) & = & x^{T}Ax-2x^{T}A\tau +\tau^{T}A\tau +\;x^{T}Lx=\;x^{T}\left(L+A\right)x-2x^{T}A\tau +\tau^{T}A\tau \\ & = & \left[x_{M}^{T}\;x_{U}^{T}\right]\left[\begin{array}{cc} S_{M} & B\\ B^{T} & S_{U} \end{array}\right]\left[\begin{array}{c} x_{M}\\ x_{U} \end{array}\right]-2\left[x_{M}^{T}\;x_{U}^{T}\right]\left[\begin{array}{cc} A_{M} & 0\\ 0 & A_{U} \end{array}\right]\left[\begin{array}{c} \tau_{M}\\ \tau_{U} \end{array}\right]+\left[\tau_{M}^{T}\tau_{U}^{T}\right]\left[\begin{array}{cc} A_{M} & 0\\ 0 & A_{U} \end{array}\right]\left[\begin{array}{c} \tau_{M}\\ \tau_{U} \end{array}\right]\\ & = & x_{M}^{T}S_{M}x_{M}+2x_{U}^{T}B^{T}x_{M}+x_{U}^{T}S_{U}x_{U}-2x_{M}^{T}A_{M}\tau_{M}-2x_{U}^{T}A_{U}\tau_{U}+\tau_{M}^{T}A_{M}\tau_{M}+\tau_{U}^{T}A_{U}\tau_{U}. \end{array}$$ (19)

We denote *L* + *A* as S, which is partitioned into marked (seed nodes) and unmarked (unseeded nodes) blocks.

$$S=\left[\begin{array}{cc} S_{M} & B\\ B^{T} & S_{U} \end{array}\right].$$ (20)

For eachcategory *c*, we define the set of labels for the seed points as a function *Q*(*v*_*j*_) = *c* for any *v*_*j*_ ∈ *V*_*M*_. Furthermore, we define a |*V*_*M*_| × 1 vector (where |·| denotes cardinality) for each category *c* at node *v*_*j*_ ∈ *V*_*M*_ as

$$m_{j}^{c}=\{\begin{array}{ll} 1 & if\;Q\left(v_{j}\right)=c,\\ 0 & if\;Q\left(v_{j}\right)\neq c. \end{array}$$ (21)

The minimization of (16), (18), (19) with respect to $x_{U}^{c}$ is given by the linear system:

Pearson distance:

$${\mathbb{P}}_{U}x_{U}^{c}=-B^{T}m^{c}+A_{U},$$ (22)

Inner product:

$$L_{U}x_{U}^{c}=-B^{T}m^{c}+A_{U}\tau_{U}^{c},$$ (23)

L2 norm square:

$$S_{U}x_{U}^{c}=-B^{T}m^{c}+A_{U}\tau_{U}^{c},$$ (24)

for one label, or

$${\mathbb{P}}_{U}X=-B^{T}M+A_{U},$$ (25)

$$L_{U}X=-B^{T}M+A_{U}T,$$ (26)

$$S_{U}X=-B^{T}M+A_{U}T,$$ (27)

for all labels, where the matrix *X* has *K* (in our problem, *K* = 2) columns for each $x_{U}^{c}$, where *M* has columns for each *m*^*c*^ and T has columns for each $\;\tau_{U}^{c}$ Therefore, we solve the probabilities matrix *X* using *K* sparse linear systems. The overall segmentation scheme is described in Table 1.

**Table 1:** Algorithm 1.

**Input**: Input image *I* and a set of foreground and background seeds
**Output**: The segmentation results.
**Step1**: Compute edge weights according to *exp*(−*β*|*g*_*i*_ − *g*_*j*_|).
**Step2**: Compute the Laplacian matrix with formula (15), the attraction propagation incidence matrix with formula (12) and the range base with formula (13).
**Step3**: Decompose the Re-built Laplacian matrix and compute the transition probability by solving the sparse linear system: $$H(X)=\{\begin{array}{c} {\mathbb{P}}_{U}X=-B^{T}M+A_{U},\left(PEA-AP\right)\\ L_{U}X=-B^{T}M+A_{U}T,\left(INN-AP\right)\\ S_{U}X=-B^{T}M+A_{U}T,\left(L2-AP\right) \end{array}$$
**Step4**: For any node *v*_*i*_, classify it as belonging to segment *k* (*k* represents the classify label) if $\;x_{U}^{k}>x_{U}^{k^{\prime }}$ for all $k^{\prime }\neq k$.
Fast Segmentation scheme with the AP

**Fig 7:**
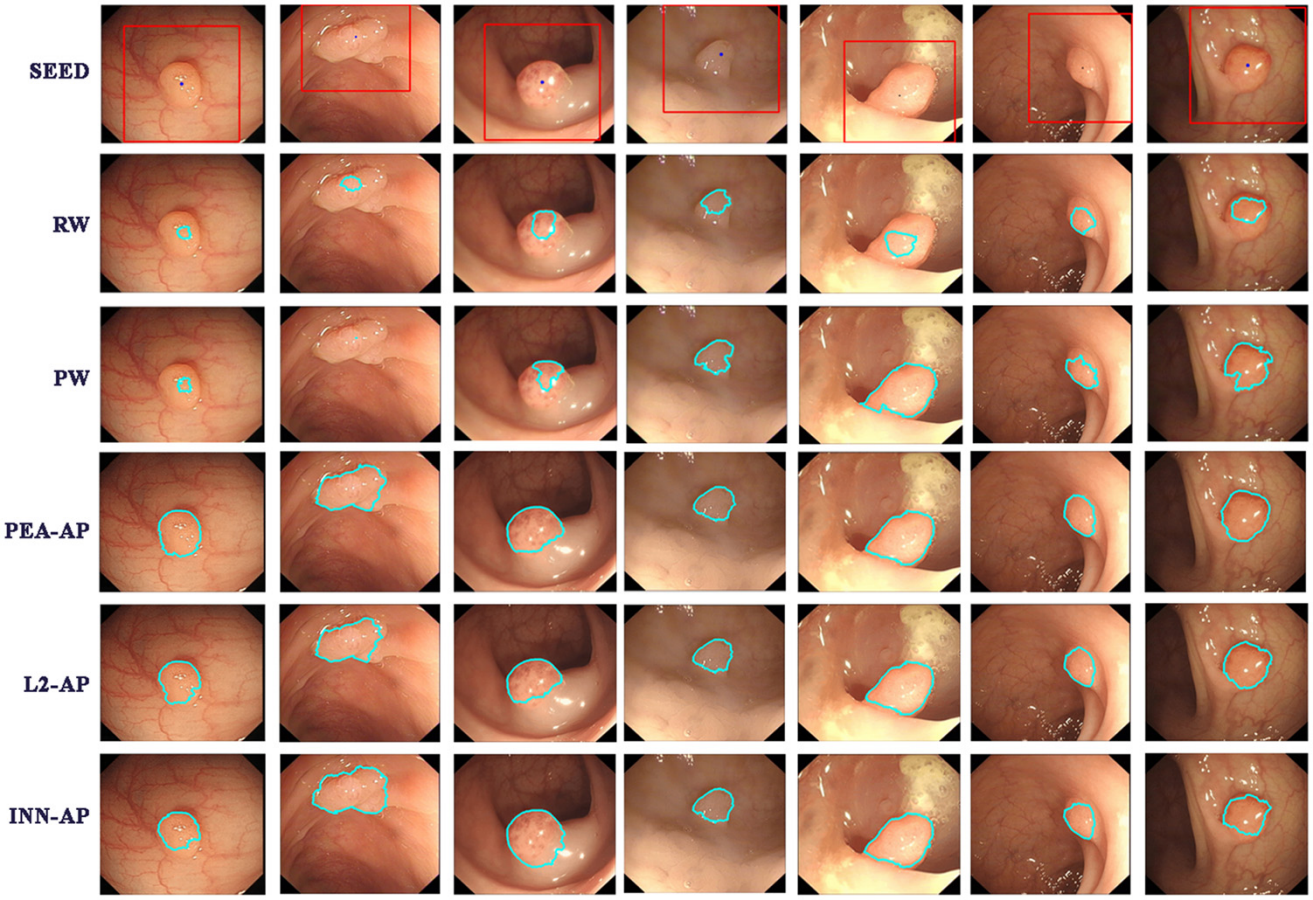
Illustration of the RW, PW and AP. The top row: seeds of foreground and background. The second row: Segmentation results obtained by the RW [34]. The third row: Segmentation results obtained by the PW [37]. The last three rows: Segmentation results obtained by the PEA-AP, L2-AP and INN-AP.

The above attraction scheme AP has many advantages and properties, such as high computational speed and robust segmentation. AP is formulated on a general graph with priori-knowledge shape, which can represent any dimension or topology, and it holds the segmentation accuracy of the abnormal region and reduces the estimates of the background region. An example of the results obtained by the AP is shown in the last three rows of Fig 7. Even when only one seed point is selected in the foreground region, the AP can improve the segmentation accuracy more effectively than other weighted graph methods. More numerical results are shown in the following Section.

## Experiments and Results

To the best of our knowledge, there are no literatures offering a large-scale analysis and validation the performance of the automatic and interactive segmentation algorithms for OC image. It is still largely unknown about whether interactive/semi-automatic methods perform better than automatic methods or vice versa. And then, almost all of the interactive method can realize automation, where the polyp was present in the center of the image (or fixed position in the image). So in this section, we present a comprehensive performance evaluation using some well-known segmentation algorithms (including automatic and interactive segmentation algorithms) for 1691 OC images with abnormal regions. In total, these images are derived from the three databases. Further details about the three different datasets are given below.

1. Database I: Database I [12] consists of 358 images, which is an open database for comprehensive assessment of polyp detection and segmentation. In this database, the ROIs define the whole area covering the polyp and they are implemented as binary images, with white masks over a black background. It is available on the website via the following link: http://mv.cvc.uab.es/projects/colon-qa/.
2. Database II: Database II [5] is a good common database that can be used to evaluate the performance of the different methods. It is made up of 533 OC images extracted from colonoscopy videos (The original dataset consists of 612 images but 79 images in them are damaged). The ground truth for the polyps consists of a mask corresponding to the region covered by the polyp in the image. This database is obtained via the following link: http://www.polyp2015.com/wp/?page_id=141.
3. Database III: Database III is obtained from the open NNUC database: http://math.nenu.edu.cn/nnucdb/, where the ground truth data are manually annotated and segmented by experts. It covers numerous types of polyp appearances, which can be used to test the performance of different segmentation methods.

In this study, the foreground seed is single and the backgroud seeds form a frame as shown in Section 2.1. And for each OC image, the same seed placement is employed by all the interactive segmentation methods. The performance of these algorithms is measured by annotated area covered (AAC) and Dice similarity coefficient (DICE) [44].

$$AAC=O_{a}/A_{a},$$ (28)

$$DICE=2O_{a}/\left(S_{a}+A_{a}\right),$$ (29)

where *S*_*a*_ is the resulting from algorithm annotation and *A*_*a*_ is an image section resulting from manual annotation. *O*_*a*_ is the number of common pixels between *S*_*a*_ and *A*_*a*_.

In our simulation, we split the data into two parts. One is for training the attraction region (AR) and the other is for testing the performance of our algorithm. Specifically, for testing Database I, we randomly choose 10% samples from Database II and Database III to compute the AR. Similarly, for testing Database II, we choose samples from Database I and Database III. And for testing Database III, we choose samples from Database I and Database II.

### Colonoscopy image segmentation on database I

**Fig 8:**
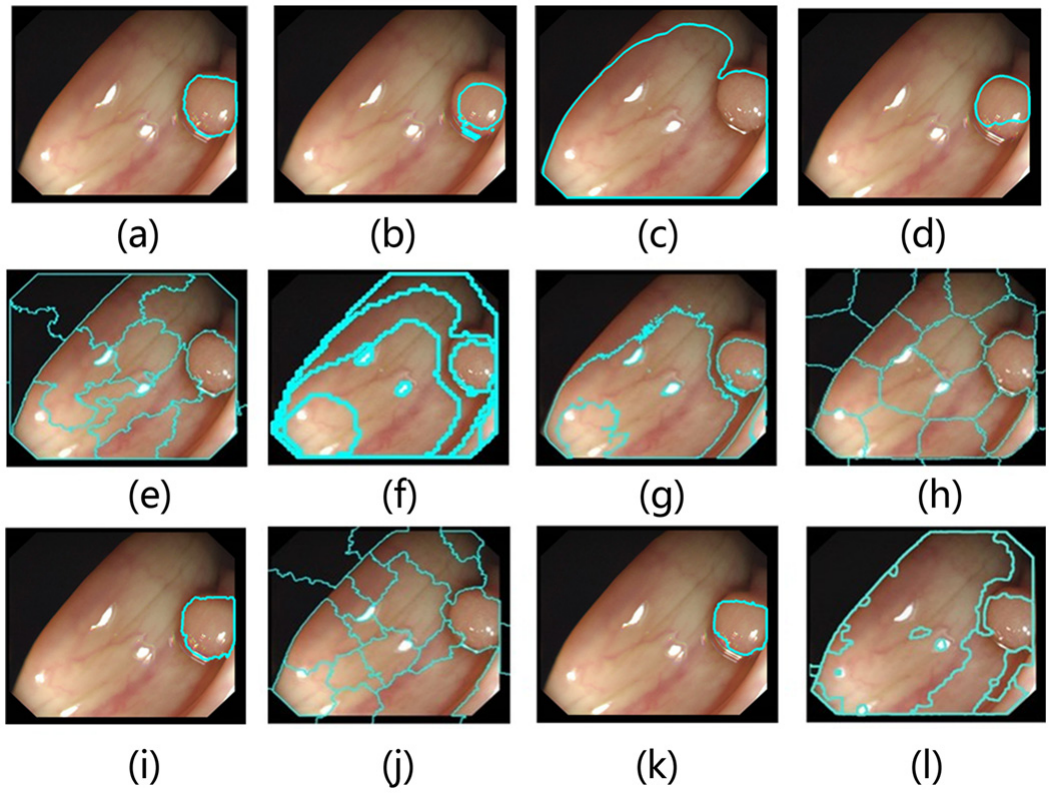
Comparison of segmentation results. (a) PEA-AP (b) ACC; (c) CV (d) DRLSE; (e) ERC; (f) FCM; (g) MS; (h) NC; (i) PW; (j) QS; (k) RW and (l) SA-DOVA.

The database I is a comprehensive data set applied in the field of colonoscopy image detection and we use it to test the proposed approaches. This database comprises 358 OC images, each of which contains 500 × 574 pixels. Fifteen related approaches are used for comparison: Active Contours for Colonoscopy (ACC) [20], Chan-Vese-Segmentation (CV) [18], Distance Regularized Level Set Evolution (DRLSE) [28], Entropy Rate Clustering (ERC) [11], Fuzzy C Means (FCM) [7], Mean Shift (MS) [8], Normalized Cuts (NC) [9], Quick Shift (QS) [10], Power watershed (PW) [37], Random Walks (RW) [34], Sector Accumulation-Depth Of Valleys Accumulation (SA-DOVA) [12], Template Matching (TM) [17] and our method. Fig 8 shows an example of the output from each method using the database I. Our method (Fig 8a) identifies the polyp edges better than other methods shown in Fig 8. The underlying reason of this result is that the attraction strategy can take advantage of the structural properties of polyp (such as geometry symmetry and local swell) to improve the precision. The output of ACC (Fig 8b) appears to be too conservative because it has high precision but it fails to identify a larger number of polyp pixels. The output of CV (Fig 8c) does not retain the shape of the original polyp. The output of DRLSE (Fig 8d) is a suitable fit for OC image segmentation but the method is too slow to be used for real-time segmentation. The output of ERC (Fig 8e) is an effective method for OC images. However, a fixed parameter is not available that works well for all OC images. The output of NC (Fig 8h) is not a bad fit in our example, but it consumes a large volume of memory. Specially, the result of PW (Fig 8i) is very well in this example, but PW is unstable and may fail in other cases, which causes a weak performance result as shown in Table 2. SA-DOVA (Fig 8l) obtains a high AAC but this method could also decrease the precision. Weak boundary of polyp is a major factor that leads to the invalidation of SA-DOVA. The other algorithms (FCM, MS, QS and RW) have various deficiencies in this case and we do not discuss them here.

**Table 2:** Comparison results of methods on database I.

Method	AAC(%)	DICE(%)
ACC[20]	63.48	54.67
CV[18]	72.80	35.34
DRLSE[28]	59.68	59.24
ERC[11]	**78.46**	61.27
FCM[7]	62.80	34.38
MS[8]	65.31	33.25
NC[9]	72.64	65.78
PW (q = 2)[37]	47.62	54.97
QS[10]	53.80	54.77
RW (*β* = 260)[34]	22.61	31.18
SA-DOVA (DVT = 0.6)[12]	61.91	55.33
SA-DOVA (DVT = 0.7)[12]	70.29	44.60
SA-DOVA (DVT = 0.8)[12]	**75.79**	36.44
TM[17]	35.75	34.89
PEA-AP (*β* = 310, *Γ* = 0.8)	69.06	**74.48**
L2-AP (*β* = 310, *Γ* = 0.8)	68.49	**74.20**
INN-AP (*β* = 340, *Γ* = 0.6)	**77.46**	**75.98**

Table 2 shows the performance of twelve algorithms on the database I according to two different measures: AAC and DICE, where the bold numbers show the top three results for each measure. In this experiment, we choose fixed parameters for our method (*β* = 310, *Γ* = 0.8; *β* = 340, *Γ* = 0.6). For algorithm ACC, CV, DRLSE, ERC, FCM, MS, NC, QS and TM, it is difficult to choose a group of fixed optimal parameters for all OC images. So we select optimal parameters for each image in order to improve the performance of these algorithms. In Table 2, DRLSE and PW get a low AAC and DICE. This result may be caused by the low quality of OC images in the database. For sparse foreground seeds, RW encounters small cut problems which cause the low values of its AAC and DICE. Algorithms FCM, MS, SA-DOVA and TM are affected by weak boundary and types of abnormal regions. Algorithms ERC, NC and AP have relatively high AAC and DICE.

### Colonoscopy image segmentation on database II

The database II comprises 533 OC images of resolution 288× 384. Fourteen approaches are used for comparison and related results are shown in the Table 3. Compared to database I, most algorithms perform better on database II for the AAC and DICE since database I contains many low quality images. Most of the algorithms keep a similar rank ordering on performance as Table 2. As shown in Table 3, both AAC and DICE for PEA-AP are in the top three. Specially, the AAC of AP is 47% better than RW, which illustrates the importance of adding the attraction propagation term since the larger AAC value means more retrieval polyp pixels. As shown in Tables 2 and 3, the DICE of general segmentation methods (such as MS, ERC and NC) is close to the SA-DOVA, ACC or TM, which are exclusively used in OC images. And the interactive methods (such as DRLSE, PW and RW) are no better than automatic methods (ERC and NC). So we intend to provide a further measure and analysis in the following Section.

**Table 3:** Comparison results of methods on database II.

Method	AAC(%)	DICE(%)
ACC[20]	74.68	52.03
CV[18]	75.88	48.26
DRLSE[28]	68.11	68.73
ERC[11]	**82.26**	74.09
FCM[7]	65.90	42.85
MS[8]	67.07	41.59
NC[9]	76.06	**76.18**
PW (q = 2)[37]	62.25	69.74
QS[10]	55.06	51.48
RW (*β* = 260)[34]	33.29	41.30
TM[17]	73.65	54.34
PEA-AP (*β* = 310, *Γ* = 0.8)	**80.54**	**80.22**
L2-AP (*β* = 310, *Γ* = 0.8)	80.13	**80.10**
INN-AP (*β* = 230, *Γ* = 0.6)	**84.06**	75.02

### Colonoscopy image segmentation on database III

**Fig 9:**
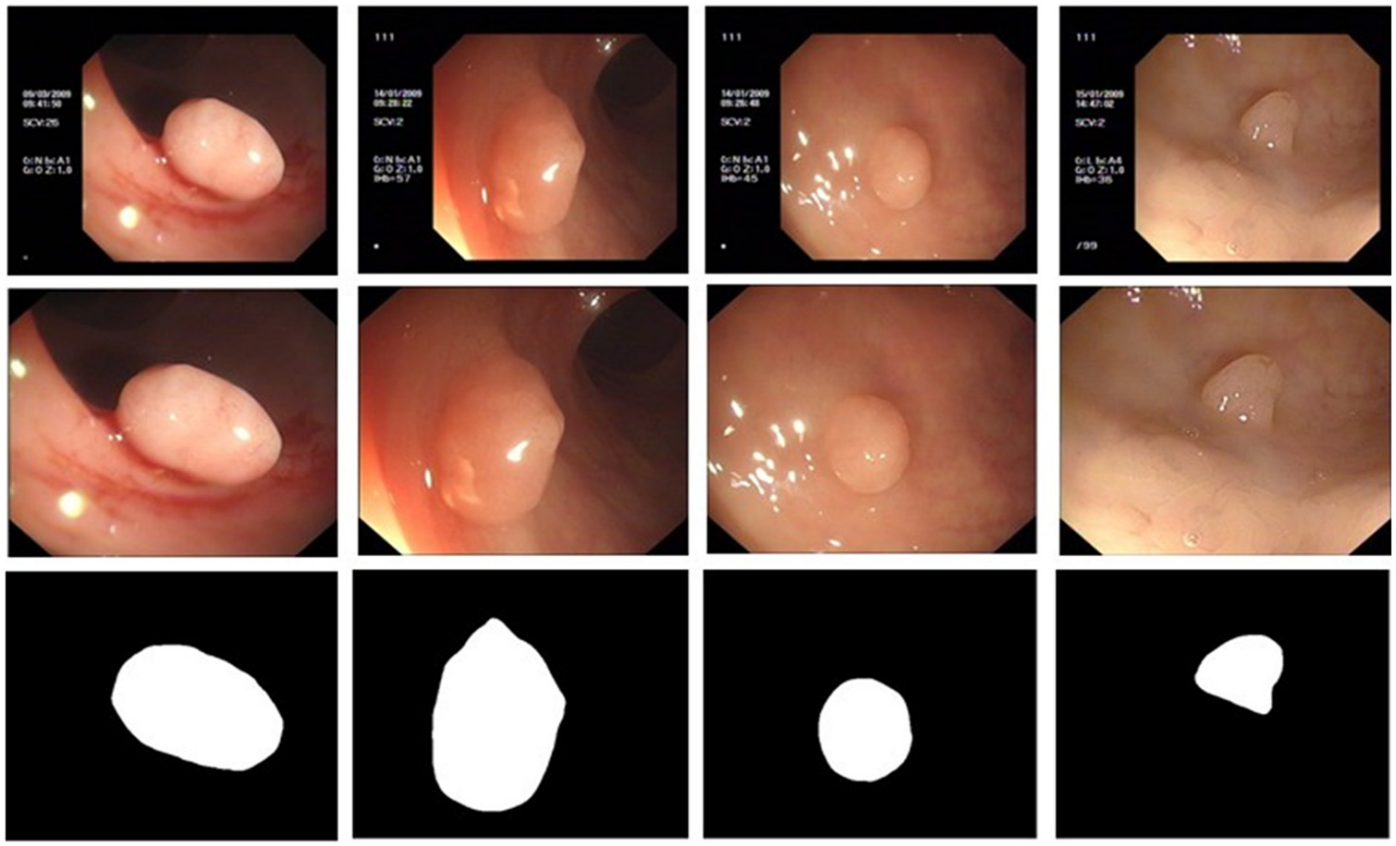
Comparison of preprocessing results. the top row: raw image; the middle row: preprocessing results; the bottom row polyp masks(the polyp is shown in white).

To promote the development of the colonoscopy image recognition field, we produce a rich set of images for researchers to analyze, i.e., an open database called the Northeast Normal University Colonoscopy (NNUC) dataset, which contains 800 OC images that cover more abnormal region types than databases I and II. The images in NNUC are derived from colonoscopy images of 400 patients, where experts print the screen after identifying potentially cancerous areas during operations. Fig 9 shows the different types of polyp appearances found in the dataset. The size of the OC images varies in different cases due to the diversity of the recording instruments employed. Thus, the areas of the images are cropped in order to reduce the effects of irrelevant information on the image segmentation process. After the cropping process, the images are resized to 408 × 474. Fig 9 shows some of the preprocess results, where the first row comprises raw images, the second row shows the preprocessing results, and the third row shows the polyp masks, which are regarded as the ground truth in the evaluation.

#### Segmentation by retrieval

To evaluate the performance of different segmentation algorithms for OC image in a more comprehensive and systematic manner, segmentation is regarded as the retrieval of polyp or non-polyp pixels. All of the segmentation methods return the designated positions of the polyps in the images as binary masks. Thus, we perform a pixel-by-pixel comparison of the polyp masks and the resulting masks to evaluate the segmentation methods. The segmentation methods assign each pixel as a polyp or a non-polyp pixel. Based on these comparisons, we obtain four possible outcomes: true positive (TP), true negative (TN), false positive (FP), or false negative (FN). We denote positive as a polyp pixel and negative as a non-polyp pixel. Thus, rather than using the two standard metrics in the evaluation, we employ six characteristic values to analyze the impact of the parameters on the segmentation results obtained with images from the NNUC database. The six characteristic values can be computed as:

Precision=TP/ (TP+FP), (30)

Recall=TP/(TP+FN), (31)

Accuracy=(TP+TN)/(TP+TN+FP+FN), (32)

Specificity=TN/(TN+FP), (33)

Fallout=FP/(TN+FP), (34)

F2measure=5×TP/(5×TP+4×FN+FP). (35)

#### Comparison of the results obtained with different segmentation algorithms

**Fig 10:**
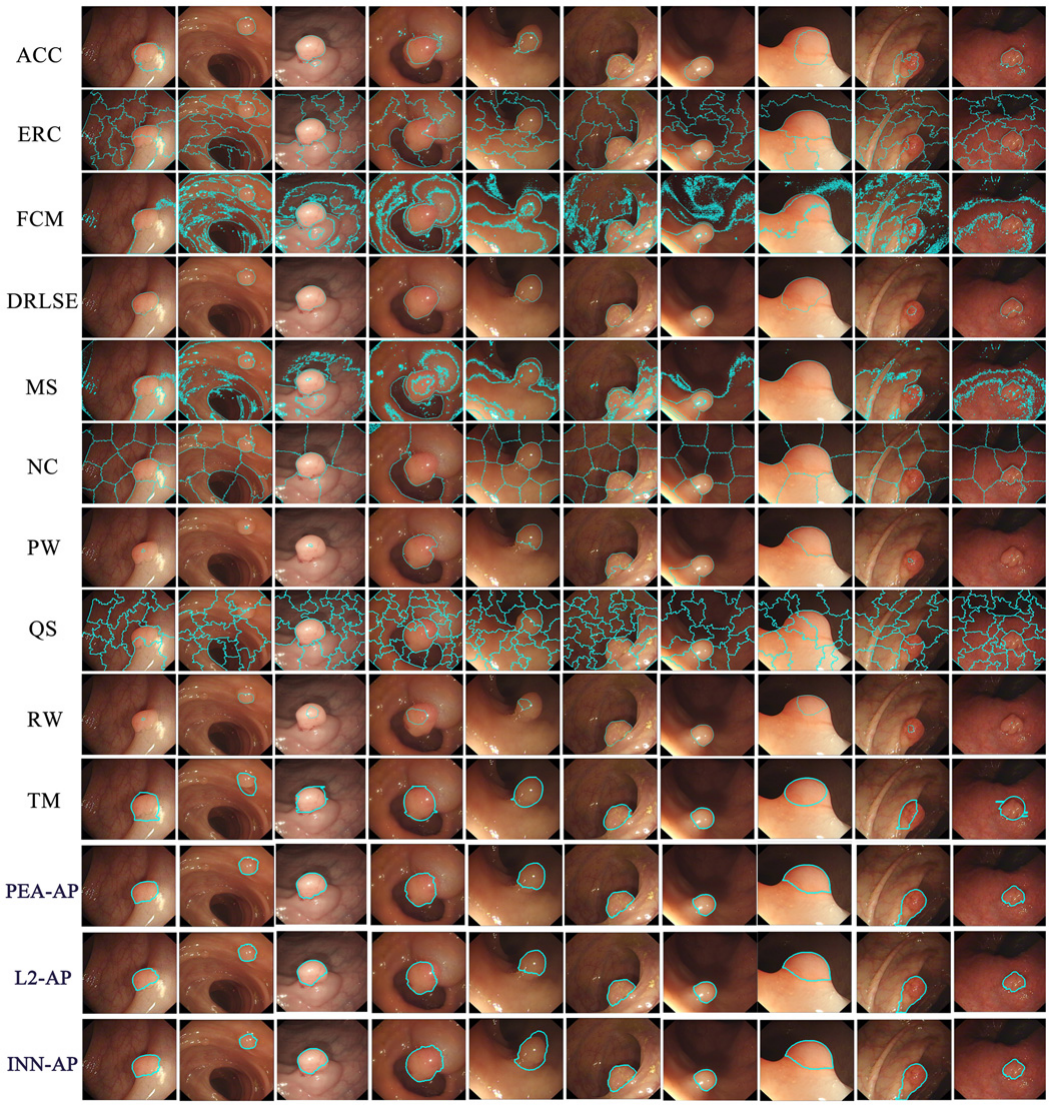
Comparison of segmentation results on the Database III.

In this section, we compare the quality of the segmentation results obtained by using the methods tested in our evaluation. Thirteen related approaches are selected for this evaluation: ACC, DRLSE, ERC, FCM, MS, NC, PW, QS, RW, TM and AP. We apply these methods to all of the 800 images in the database III. Fig 10 shows some results of the output from each method using image from the database III.

The optimal segmentation results are presented in Table 4, where the bold numbers show the top three scores for each characteristic. As shown in Table 4, among all the algorithms tested, our AP method achieves the highest Accuracy, Recall, F2measure, and DICE. It has seven characteristics in the top three. Although the Precision with AP is less than that with some other algorithms, the Recall (according to [44], the value of AAC is the same as that of Recall) is 22% better compared with PW. The DICE of AP is 28% better compared with that of RW. This demonstrates that our method can find more polyp pixels with small errors, and has greater flexibility for adapting to variation in the shape of objects.

**Table 4:** Comparison results of methods on database III.

Method	Accuracy(%)	DICE(%)	F2measure (%)	Fallout	Precision (%)	Recall/(%)	Specificity(%)
ACC[20]	95.52	65.08	66.62	0.0253	67.29	69.26	97.47
DRLSE[28]	95.91	68.58	67.93	0.0272	68.53	81.63	97.28
ERC[11]	**96.89**	74.44	78.61	0.0209	72.18	**84.07**	97.91
FCM[7]	79.60	34.02	47.98	0.2052	24.12	74.10	79.48
MS[8]	82.95	34.27	43.37	0.1581	28.35	60.99	84.19
NC[9]	96.53	**75.24**	75.96	0.0222	77.08	77.59	97.78
PW (q = 2)[37]	96.48	64.56	60.81	**0.0096**	**91.89**	60.23	**99.04**
QS[10]	91.86	30.07	31.53	0.0411	34.64	35.03	95.89
RW (*β* = 260)[34]	95.38	45.30	39.78	**0.0023**	**97.40**	37.45	**99.77**
TM[17]	96.45	69.21	70.90	0.0139	75.32	73.87	98.61
PEA-AP (*β* = 310, *Γ* = 0.8)	**97.51**	**81.44**	**81.50**	0.0108	85.67	**83.38**	98.92
L2-AP (*β* = 310, *Γ* = 0.8)	**97.52**	**81.49**	**81.28**	**0.0102**	**86.19**	82.89	**98.98**
INN-AP (*β* = 230, *Γ* = 0.6)	95.69	73.98	**79.29**	0.0354	72.83	**87.82**	96.46

### Comparison of the processing time

**Fig 11:**
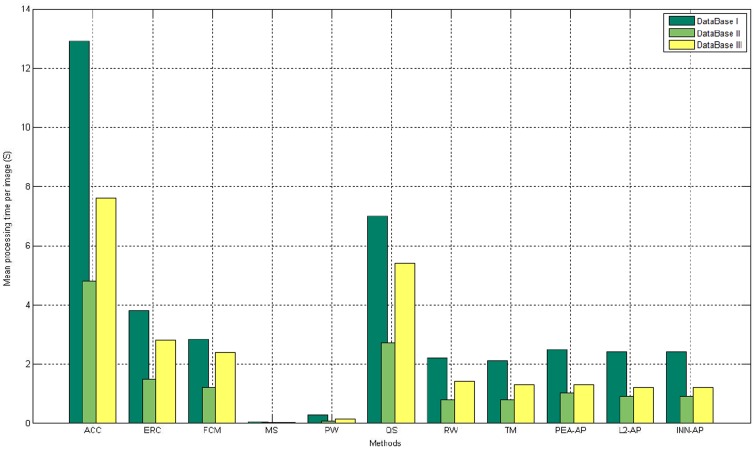
Mean processing time per OC image on each database.

We compare the time costs of various algorithms in Fig 11. The experiments are performed by using Matlab R2009b on an Intel Core(TM)2 Duo CPU with 2.93 GHz and 2 GB memory except PW (PW uses the available C++ libraries for operations on Linux-OS). As shown in Fig 11, MS is the fastest method, which required 0.02–0.03s, but its performance is fair. The solution times are slow with ACC and QS, i.e., 4.8–12.9s and 5–7s. The processing time with AP is close to that with RW, it costs 0.79–2.6s to calculate the solution for each image. This indicates that our method can be used for online processing.

## Discussion

In the proposed method, there are two parameters, a weighted parameter ***β*** and a probability threshold ***Γ***, to control the performance of segmentation algorithms. We discuss the sensitivity on the two parameters for the proposed method, and use the same initialization strategy for processing each OC images. The five characteristic values can be used to compare the robustness of our method. It is because that the Recall is the ratio of correctly detected polyp pixels to all polyp pixels. High values indicate a high number of pixels available for classification of the polyp. The Precision is the number of correctly detected polyp pixels divided by all polyp pixels. A high value indicates a low number of polyp pixels incorrectly regarded as non-polyp pixels. The accuracy is the fraction of all pixels which were classified correctly. The specificity is the number of correctly detected non-polyp pixels divided by all non-polyp pixels. The F2measure is complementary metric on segmentation performance and it combines Precision and Recall into an only measure.

**Fig 12:**
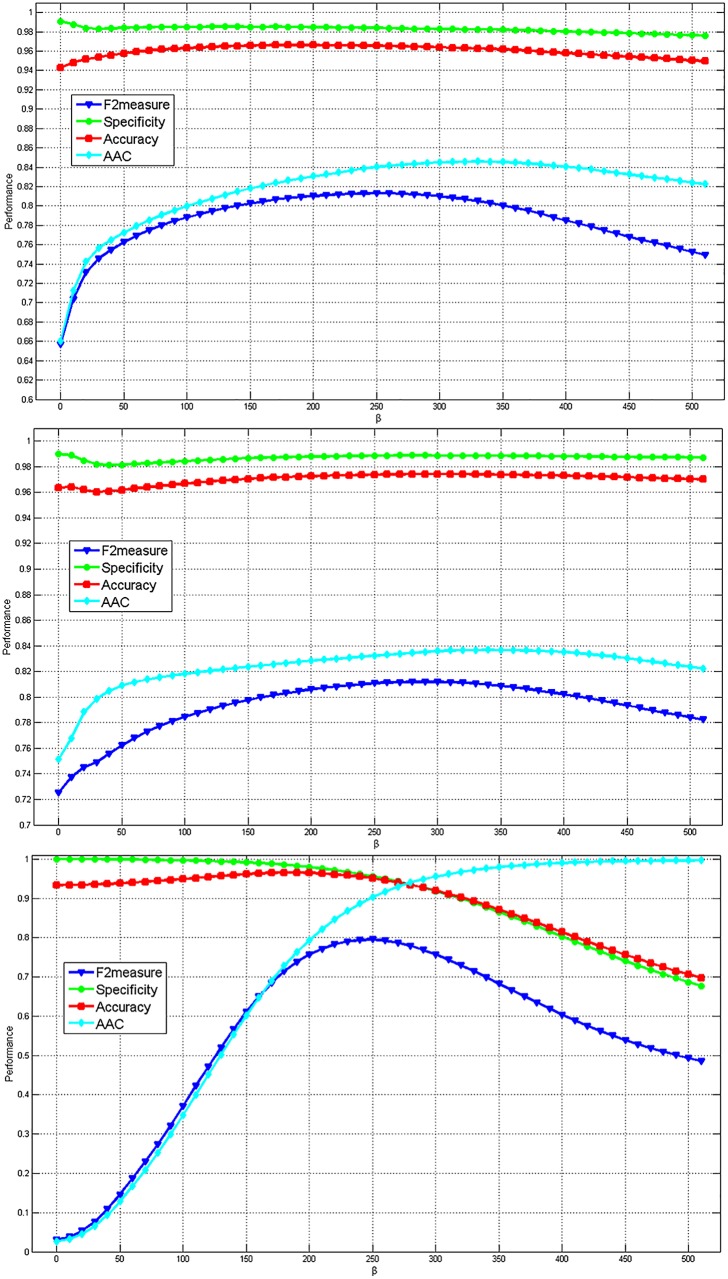
The average quantitative evaluation of the segmentation results by varying the value of *β* in5-fold validation. (a) PEA-AP (b) L2-AP (c) INN-AP.

**Fig 13:**
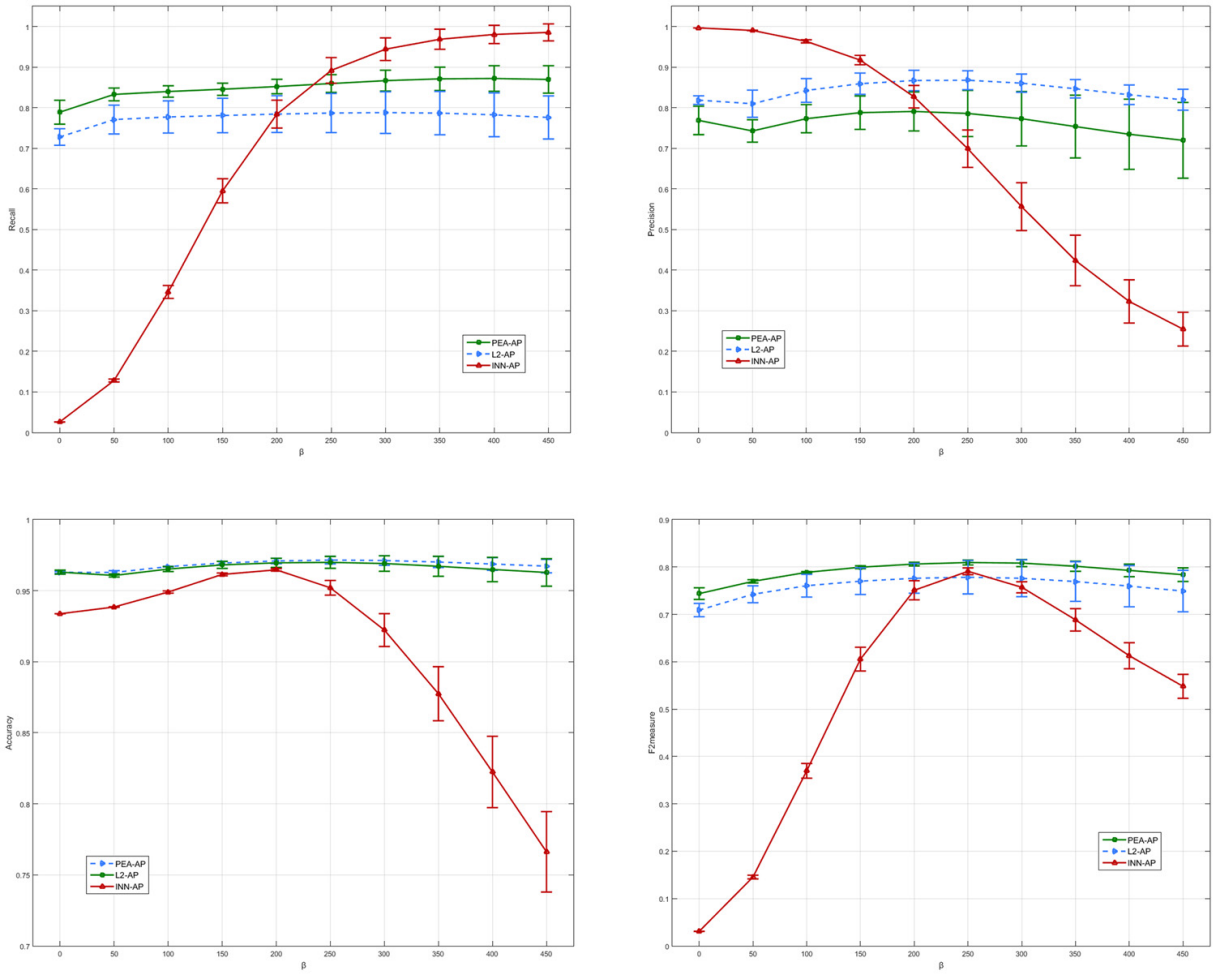
The average quantitative evaluation of the AP results by varying the value of *β* and *Γ* in5-fold validation.

First, we consider the performance of our algorithms based on various weighted parameter ***β***. Fig 12 shows quantitative evaluation of the segmentation results with ***Γ*** = **0.7** in 5-fold validation. It can be seen that the Accuracy and the Specificity obtained by PEA-AP and L2-AP are not sensitive as ***β*** increases. Although the AAC is 0.70–0.82 and the F2measure is 0.73–0.79, the performance of the proposed scheme is acceptable. Also we note that INN-AP is sensitive as ***β*** increases in the Fig 12. This is because that compared to two other algorithms, the INN-AP only considers the shift operation for boosting or inhibiting the probability. Next, we vary the parameters ***β*** and ***Γ*** at the same time. The experiment results on the Precision, Recall, Accuracy and F2measure are shown in Fig 13. For PEA-AP and L2-AP, the Accuracy is large than 95%. The average AAC and F2measure are roughly from 0.70 to 0.80. This means that these two algorithms are robust for parameter selection.

## Conclusion

In this study, we propose a flexible framework for interactive image segmentation in OC image segmentation. Compared with other segmentation methods, our new framework AP provides an efficient, real-time, robust solution. We introduce the shape probability region and the attractive strategy, which allow our AP algorithm to start with only one foreground seed, and automatically detect the contours of polyps. After adjusting the threshold parameter, the AP algorithm can restrain the transition probabilities of pixels in non-polyp regions without any pre-processing of the raw OC images. Moreover, it can effectively improve the accuracy of the segmentation of polyp regions, although they are very noisy with uneven brightness.

In order to evaluate the performance of our segmentation methods, we built an open database of 800 OC images that contained multiple types of abnormal regions. Furthermore, we use seven characteristics to compare the quality of various algorithms, and we demonstrated the processing efficiency with various database. Our experimental results show that AP algorithm is effective and practical for polyp segmentation in colonoscopy images.

## References

[1] Brenner H, Kloor M, Pox CP. Colorectal cancer. The Lancet 2014; 383: 1490–1502. 10.1016/S0140-6736(13)61649-9 24225001

[2] Stock C, Hoffmeister M, Birkner B, Brenner H. Inter-physician variation in follow-up colonoscopies after screening colonoscopy. PLoS ONE 2013; 8(7):e69312 10.1371/journal.pone.0069312 23874941 PMC3715496

[3] Segnan N, Patnick J, Karsa LV. European guidelines for quality assurance in colorectal cancer screening and diagnosis. Publications Office of the European Union Luxembourg 2011.

[4] Tresca A. The stages of colon and rectal cancer. New York Times 2010.

[5] Bernal J, Sánchez J, Esparrach GF, Gil D, Miguel CR, Vilariño F. WM-DOVA maps for accurate polyp highlighting in colonoscopy: validation vs. saliency maps from physicians. Computerized Medical Imaging and Graphics 2015; 43: 99–111. 10.1016/j.compmedimag.2015.02.007 25863519

[6] Huang Q, Yang F, Liu L, Li X. Automatic segmentation of breast lesions for interaction in ultrasonic computer-aided diagnosis. Information Sciences 2015; 314: 293–310.

[7] Bezdek JC. Pattern recognition with fuzzy objective function algorithms. Plenum Press; 1981.

[8] Comaniciu D, Meer P. Mean shift: a robust approach toward feature space analysis. IEEE Trans. on Pat. Anal. and Mach. Int. 2002; 24: 603–619.

[9] Shi J, Malik J. Normalized cuts and image segmentation. IEEE Trans. on Pat. Anal. and Mach. Int. 2000; 22: 888–905.

[10] Vedaldi A, Soatto S. Quick shift and kernel methods for mode seeking. in Proc. of ECCV’08 2008; 5305: 705–718.

[11] Liu MY, Tuzel O, Ramalingam S, Chellappa R. Entropy-rate clustering: cluster analysis via maximizing a submodular function subject to a matroid constraint. IEEE Trans. on Pat. Anal. and Mach. Int. 2014; 36: 99–112. 10.1109/TPAMI.2013.107 24231869

[12] Bernal J, Sánchez J, Vilariño F. Towards automatic polyp detection with a polyp appearance model. Pattern Recognition 2012; 45: 3166–3182.

[13] Ferrucci JT. Colon cancer screening with virtual colonoscopy: promise, polyps, politics, Am. J. Roent. 2011; 177: 975–988. 10.2214/ajr.177.5.1770975 11641151

[14] Li P, Chan KL, Krishnan SM. Learning a multi-size patch-based hybrid kernel machine ensemble for abnormal region detection in colonoscopic images. in Proc. of CVPR’05 2005; 2: 670–675.

[15] Ravesteijn VFV, Wijk CV, Vos FM, Truyen R, Peters JF, Stoker J, et al Computer-aided detection of polyps in CT colonography using logistic regression. IEEE Trans. on Medical Imaging 2010; 29: 120–131. 10.1109/TMI.2009.2028576 19666332

[16] Stehle T, Auer R, Gross S, Behrens A, Wulff J, Aach T, et al Classification of colon polyps in NBI endoscopy using vascularization features Medical Imaging 2009: Computer-Aided Diagnosis, Orlando, SPIE 2009; 7260.

[17] Gross S, Kennel M, Stehle T, Wulff J, Tischendorf J, Trautwein C, et al Polyp segmentation in NBI colonoscopy Bildverarbeitung für die Medizin 2009, Springer, Berlin 2009; 252–256.

[18] Breier M, Gross S, Behrens A. Chan-Vese-segmentation of polyps in colonoscopic image data. in: Proceedings of the 15th International Student Conference on Electrical Engineering POSTER 2011.

[19] Cong Y, Wang S, Liu J, Cao J, Yang Y, Luo J. Deep sparse feature selection for computer aided endoscopy diagnosis. Pattern Recognition 2015; 48: 907–917.

[20] Breier M, Gross S, Behrens A, Stehle T, Aach. T. Active contours for localizing polyps in colonoscopic NBI image data. Medical Imaging 2011: Computer-Aided Diagnosis. SPIE 7963. 2011.

[21] Chan TF, Vese LA. Active contours without edges. IEEE Trans. on Image Processing 2001; 10: 266–277. 10.1109/83.902291 18249617

[22] Huang C, Zeng L. An active contour model for the segmentation of images with intensity inhomogeneities and bias field estimation. PLoS ONE 2015; 10(4): e0120399 10.1371/journal.pone.0120399 25837416 PMC4383562

[23] Nejati JA, Unsworth CP, Graham ES. A cell derived active contour (CDAC) method for robust tracking in low frame rate, low contrast phase microscopy—an example: the human hNT astrocyte. PLoS ONE 2013; 8(12): e82883 10.1371/journal.pone.0082883 24358233 PMC3866173

[24] Wang Y, Zhu C, Zhang J, Jian Y. Convolutional virtual electric field for image segmentation using active contours. PLoS ONE 2014; 9(10): e110032 10.1371/journal.pone.0110032 25360586 PMC4216009

[25] Li Y, Sun J, Tang CK, Shum HY. Lazy snapping. in ACM Siggraph 2004; 23(3): 303–308.

[26] Adalsteinsson D, Sethian JA. The fast construction of extension velocities in level set methods. J. Comput. Phys. 1999; 148: 2–22.

[27] Konukoglu E, Acar B, Paik DS, Beaulieu CF, Rosenberg J, Napel S. Polyp enhancing level set evolution of colon wall: method and pilot study. IEEE Trans. on Medical Imaging 2007; 26: 1649–1656. 18092735 10.1109/tmi.2007.901429

[28] Li C, Xu C, Gui C, Fox MD. Distance regularized level set evolution and its application to image segmentation. IEEE Trans. on Image Processing 2010; 19: 3243–3254. 10.1109/TIP.2010.2069690 20801742

[29] Paragios N, Deriche R. Geodesic active contours and level sets for detection and tracking of moving objects. IEEE Trans. on Pat. Anal. and Mach. Int. 2000; 22: 266–280.

[30] Boykov Y, Funka-lea G. Graph cuts and efficient N-D image segmentation. Int. J. Comput. Vision 2006; 70: 109–131.

[31] Boykov Y, Kolmogorov V. An experimental comparison of mincut/max-flow algorithms for energy minimization in vision. IEEE Trans. on Pat. Anal. and Mach. Int. 2004; 26: 1124–1137. 10.1109/TPAMI.2004.60 15742889

[32] Rother C, Kolmogorov V, Blake A. GrabCut: Interactive foreground extraction using iterated graph cuts. In ACM Siggraph 2004; 23 (3): 307–312.

[33] Doyle P, Snell L. Random walks and electric networks, ser carus mathematical monographs. washington, D.C.: Mathematical Association of America 1984; 22.

[34] Grady L. Random walks for image segmentation. IEEE Trans. on Pat. Anal. and Mach. Int. 2006; 28: 1768–1783. 10.1109/TPAMI.2006.233 17063682

[35] Grady L, Schiwietz T, Aharon S, Westermann R. Random walks for interactive organ segmentation in two and three dimensions: implementation and validation, in Proceedings of MICCAI 2005 II, ser. LCNS, Duncan J. and Gerig G., Eds., MICCAI Society. Palm Springs, CA: Springer 2005; 3750: 773–780. 10.1007/11566489_95 16686030

[36] Bai X, Sapiro G. A geodesic framework for fast interactive image and video segmentation and matting in Proc. of ICCV’07 2007; 1–8.

[37] Couprie C, Grady L, Najman L, Talbot H. Power watershed: a unifying graph-based optimization framework. IEEE Trans. on Pat. Anal. and Mach. Int. 2011; 33: 1384–1399. 10.1109/TPAMI.2010.200 21079274

[38] Sinop AK and Grady L. A seeded image segmentation framework unifying graph cuts and random walker which yields a new algorithm In Proc. of ICCV’07 2007; 1–8.

[39] Harary F. Graph theory. Addison-Wesley; 1994.

[40] Aslan MS, Ali A, Chen DQ, Arnold B, Farag AA, Xiang P. 3D vertebrae segmentation using graph cuts with shape prior constraints in Proc. of ICIP 2010; 2193–2196.

[41] Chowdhury AS, Rudra AK, Sen M, Elnakib A, El-Baz A. Cerebral white matter segmentation from MRI using probabilistic graph cuts and geometric shape priors in Proc. of ICIP 2010; 3649–3652.

[42] Grosgeorge D, Petitjean C, Dacher JN, Ruan S. Graph cut segmentation with a statistical shape model in cardiac MRI, Computer Vision and Image Understanding 2013; 117:1027–1035.

[43] Ciurte A, Bresson X, Cuisenaire O, Houhou N, Nedevschi S, Thiran JP, et al Semi-supervised segmentation of ultrasound images based on patch representation and continuous min cut. PLoS ONE 2014; 9(7): e100972 10.1371/journal.pone.0100972 25010530 PMC4091944

[44] Riaz F, Ribeiro MD, Coimbra MT. Quantitative comparison of segmentation methods for in-body images. In: Annual International Conference of the IEEE Engineering in Medicine and Biology Society 2009; 5785–5788. 10.1109/IEMBS.2009.5332540 19963659

